# Cutaneous Infection Associated With Myroides odoratimimus Bacteremia in a Diabetic Patient

**DOI:** 10.7759/cureus.41328

**Published:** 2023-07-03

**Authors:** Nataly Echevarría-Castro, Andrea Matayoshi-Pérez, Kevin Angel Silva-Parra, Danitza Rojo-García, Carolina Cucho Espinoza

**Affiliations:** 1 Medical Education, Peruvian University of Applied Sciences, Lima, PER; 2 Medicine, Universidad Peruana de Ciencias Aplicadas, Lima, PER; 3 Internal Medicine, Hospital Nacional Dos de Mayo, Lima, PER; 4 Pathology and Laboratory Medicine, Hospital Nacional Dos de Mayo, Lima, PER; 5 Medical Education, Universidad Ricardo Palma, Lima, PER

**Keywords:** peru, diabetes type 2, antibiotic resist, gram-negative bacteremia, myroides odoratimimus

## Abstract

In daily medical practice, there exist multidrug resistance bacteria that are not widely recognized. One example of that is the *Myroides spp.*, a Gram-negative bacillus causing skin, urinary, and bloodstream infections, especially in immunocompromised patients. In recent years, multiple cases of difficult hospital management have been reported. Currently, there are no specific guidelines for the prevention and treatment of this infection. This case report presents a patient with type 2 diabetes mellitus with a severe skin infection caused by this microorganism. This is the first case report in Peru of a severe skin infection related to *Myroides odoratimimus* bacteremia.

## Introduction

*Myroides spp.* are obligate aerobic, non-fermenting, non-motile Gram-negative bacilli with a characteristic yellow stain and odor [1]. Species with clinical relevance are *M. odoratus*, *M. odoratimimus*, *M. injenensis*, and *M. phaeus* [2]. They are ubiquitous microorganisms and, formerly, considered of low pathogenicity [2]. However, in recent years, multiple cases associated with *M. odoratus* or *M. odoratimimus* have been reported, causing skin, urinary, and bloodstream infections in immunocompromised patients, which have generated concern in the hospital environment due to the difficult management of the infection [3-6]. It has been observed that *Myroides spp.* contains various genes that lead to the appearance of antibiotic multiresistance, like b lactamase production gene (MUS-1, TUS-1, bla-OXA-347, bla-OXA-209), tetracicline resistance gene (tetX), chloranfenicol resistance gene (cat), intracellular surviving gene (katA, clpP, Ef-Tu, and sodB), iron competing gene (DnaK, Hsp60), and biofilm production genes [3, 6-10]; in addition, they possess virulence factors that allow their rapid dissemination and destruction of host tissue [11], becoming a disease with high morbidity and mortality. We discuss first reported case of *M. odoratimimus* pathogen in a newly diagnosed diabetic and drug-abused patient in Peru. 

## Case presentation

A 43-year-old male patient, obese, with a previous history of essential hypertension, came to the emergency room due to a 21-day progressive lesion on the upper lip associated with increased swelling, limited mouth opening, and intense pain that does not subside with anti-inflammatories. He denied fever, oral intolerance, and visual or smell alteration. A family member refers to unhealthy living conditions, recreational marijuana, and cocaine use. Denies other chronic illnesses or previous hospitalization.

**Figure 1:**
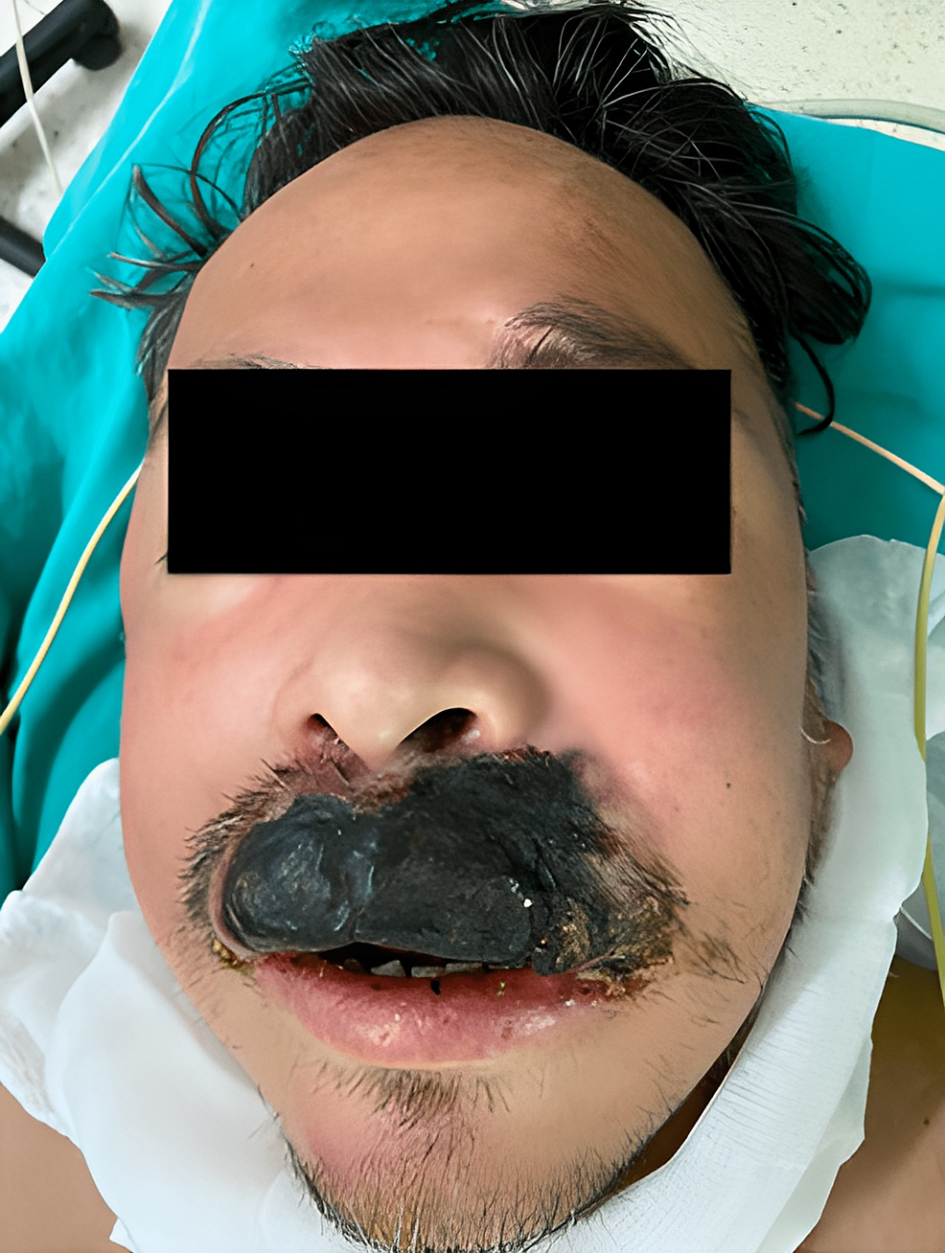
Patient at the time of admission to EMG. A necrotic ulcerated lesion of approximately 6 cm x 4 cm x 1 cm with defined edges on the upper lip is present. EMG, emergency

The patient was conscious, afebrile, and oriented with hemodynamically stable vital signs. A necrotic ulcerated lesion of approximately 6 cm x 4 cm x 1 cm with well-defined borders on the upper lip that does not compromise the hard palate, teeth, nasal cartilage, or bone structures is evident. There is a fetid odor accompanied by brown discharge and considerable facial edema (Figure 1). 

The blood test results on admission are specified in Table 1. A contrast-enhanced tomography of the facial mass was performed, revealing thickening of the soft palate and sclerosis of the upper jaw without lytic involvement. There is no evidence of cavernous sinus involvement. Due to the patient's epidemiological and clinical characteristics, the diagnosis of invasive mucormycosis plus secondary bacterial skin infection was considered. Empirical antibiotic therapy was started with oxacillin 2 g intravenously (IV) every 4 h plus clindamycin 600 mg IV every 8 h after obtaining four serial blood cultures to search for fungi and common germs; hyperglycemic therapy with insulin R was started. 

**Table 1:** Blood test result on admission.

Blood test item	Value
Glucose	515 mg/dL
HbA1c	8.2%
Leukocytes	11,090 cells/mm3
Neutrophils	9,537 cells/mm3 with left deviation
C-reactive protein	421 mg/dL
Creatinine	0.9 mg/dL
HIV rapid test	Negative

**Figure 2:**
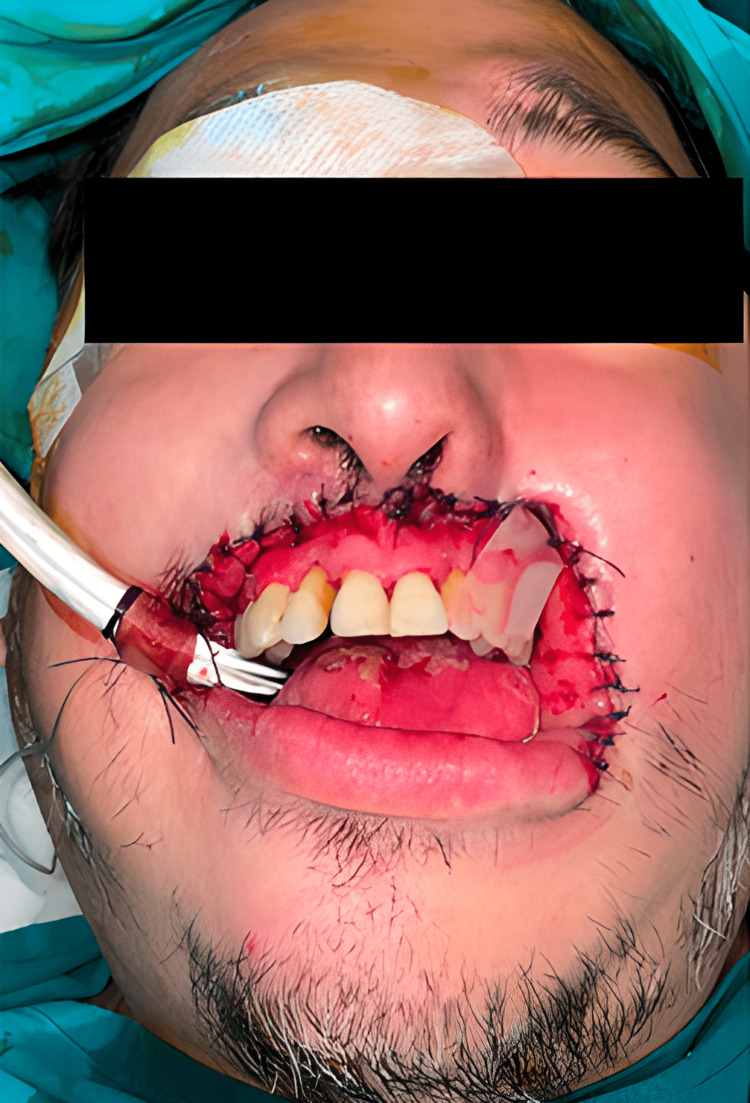
Face of the patient after surgical cleaning and extraction of the upper lip.

Surgery was performed 10 h after the patient was admitted. A 6 cm x 3 cm necrotic lesion that occupies the full thickness of the upper lip and left nose extended to the bilateral labial commissure with purulent content with a bad odor was reported. Friable whitish tissue with purulent content was discovered in the subcutaneous tissue and left cheek. There was no involvement of the bone tissue. Edema was observed on the left side of the face, including the eyelid and left cheek. The oral cavity did not exhibit any ulcers but showed signs of oral thrush (Figure 2). A frozen biopsy was performed intraoperatively showing structures compatible with fungal and bacterial infection. Tissue samples were obtained for anatomopathological study and culture of secretion and surgical region for fungi and bacteria was sent. Antifungal treatment was started with amphotericin deoxycholate 70 mg every 24 h.

On the third hospital day, the patient had a declining clinical picture, and hence antibiotic therapy was switched to broad-spectrum antibiotics namely, meropenem 1 g IV every 8 h, vancomycin 1 g IV every 12 h, and isavuconazole 372 mg IV every 8 h waiting for culture results. Vancomycin levels were not followed.

*Myroides odoratimimus* was isolated from the four blood cultures, the blood cultures which were positive at 13 h (Table 2). The biopsy result was compatible with severe inflammation secondary to extracellular bacterial proliferation. On the fifth day of hospitalization, the patient presented severe septic symptoms without meningeal involvement associated with seizures, causing the patient's death. Before the deceases, a new set of blood cultures was obtained, which subsequently revealed the growth of *M. odoratimimus*.

**Table 2:** Antibiogram Myroides odoratimimus multidrug resistant.

Antibiotic	Minimum inhibitory concentration	Interpretation
Amikacin	>32	Resistant
Cefepime	>16	Resistant
Ceftazidime	>16	Resistant
Ciprofloxacin	>2	Resistant
Gentamicin	>8	Resistant
Imipenem	8	Intermediate
Levofloxacin	>4	Resistant
Piperacillin-Tazobactam	64/4	Intermediate
Trimethoprim-Sulfamethoxazole	>2/38	Resistant

## Discussion

Currently, there are fewer than 100 case reports for *M. odoratimimus*; most of these describe skin infections, urinary tract infections, or bacteremia associated with severe septic symptoms and serious illness [10, 12]. Similar to what was found in our patient, highlights the association with states of immunosuppression [10, 12]. In a case report in Colorado, a patient with a history of diabetes mellitus developed severe bacteremia secondary to a skin lesion [13]. In another report, a soft tissue infection in a patient with a chronic ulcerative lesion led to a right supramalleolar amputation [14]. Finally, a case series reaffirms the relationship between a state of immunosuppression and the appearance of a skin infection by Myroides spp [5].

Within the differential diagnosis of a blackish scar on the face are deep fungal infections [15], such as facial mucormycosis, a rare disease with high mortality caused by fungi of the order Mucorales [16]. There is a strong association between this disease and uncontrolled diabetes mellitus [16], which is why it was initially believed to be the patient's diagnosis. However, other possible etiologies should not be forgotten, such as ecthyma which is a bacterial infection that causes a chronic blackish ulcerous lesion that can compromise deep planes [15]. The best way to determine the origin of skin pathologies is a microbiological and anatomopathological study [11], tests carried out on the patient to find the definitive diagnosis.

The microbiological diagnosis of *M. odoratimimus* was made using the identification panel of the Phoenix M50 equipment (Becton Dickinson, Franklin Lakes, NJ) whose isolation was obtained in MacConkey agar at 24 h under aerobic conditions, which was corroborated with the MALDI TOF mass spectrometry methodology (matrix-assisted ionization-desorption with time of flight) in the Microflex LT/SH BD equipment (Bruker Daltonics, Billerica, MA).

The virulence factors of *Myroides spp.* are alarming, highlighting their ability to generate biofilms and the antibiotic resistance genes to beta-lactams, carbapenems, aztreonam, and quinolones, among others [6, 11-12]. In this case, the patient initially received oxacillin, clindamycin, and amphotericin B due to suspicion of secondary fungal and bacterial infection; however, when it did not respond favorably, it was rotated to meropenem, vancomycin, and isavuconazole. Antibiogram results showed multidrug resistance and intermediate sensitivity only to imipenem and piperacillin-tazobactam. In reported cases of soft tissue infection and osteomyelitis, isolated species of multidrug-resistant Myroides responded favorably with meropenem or imipenem [2, 6, 9]. In a hospital outbreak in Romania of urinary infections caused by multiresistant *Myroides spp.*, patients responded favorably to minocycline [7]. However, in another outbreak of intrahospital urinary infections, patients who received meropenem-colistin and tigecycline, respectively, died [8]. Although in most cases a favorable evolution was observed, despite the multiple resistance of *Myroides spp.*, resistant species have also been reported, even to antibiotics such as piperacillin, tazobactam, imipenem, meropenem, and levofloxacin, used in the aforementioned reports [5]. Unfortunately, in the case presented, despite the 5 days duration of the antibiotic treatment, the posterior rotation of medications with the results of the antibiogram and the preserved kidney function, the patient had an unfavorable evolution and a fatal outcome. This is the first case report in Peru of a severe skin infection related to *M. odoratimimus* bacteremia.

## Conclusions

Infections secondary to *Myroides spp.* remain poorly understood. The unfavorable outcome in immunocompromised patients and the high bacterial resistance of this pathogen generate the need to explore resistance mechanisms, virulence factors, and therapeutic alternatives. It is important to highlight the importance of a comprehensive evaluation considering risk factors and health determinants to arrive at a timely diagnosis and work plan, promote effective treatment and improve outcomes in our patients.
